# Clinical characteristics and short-term recovery of hyposmia in hospitalized non-severe COVID-19 patients with Omicron variant in Shanghai, China

**DOI:** 10.3389/fmed.2022.1038938

**Published:** 2022-11-07

**Authors:** Jun Shen, Li Wu, Ping Wang, Xiaolei Shen, Yuhan Jiang, Jianren Liu, Wei Chen

**Affiliations:** Department of Neurology, Shanghai Ninth People's Hospital, Shanghai Jiao Tong University School of Medicine, Shanghai, China

**Keywords:** COVID-19, Omicron, hyposmia, IL-6, recovery

## Abstract

**Background:**

Olfactory dysfunction is a common neurological symptom of Corona Virus Disease 2019(COVID-19). Little is known about hyposmia after COVID-19 infection with Omicron variant in Chinese population.

**Objective:**

To investigate the incidence, clinical characteristics and recovery of hyposmia in hospitalized non-severe COVID-19 patients with Omicron variant in Shanghai, China.

**Methods:**

Three hundred and forty-nine Chinese non-severe COVID-19 patients with Omicron variant were consecutively enrolled in a designated hospital to investigate the incidence of hyposmia in hospitalization and the recovery rate 1 month later. The visual assessment scale (VAS) was used to evaluate the severity of hyposmia. We compared the demographic, clinical features and treatment outcomes, as well as laboratory parameters between patients with and without hyposmia.

**Results:**

The cross-sectional survey showed that 22 (6.3%) hospitalized patients with non-severe COVID-19 had hyposmia. Patients with hyposmia were younger (61.5 vs. 72.0, *p* = 0.002), had more related clinical symptoms (sore throat, cough, poor appetite, diarrhea, myalgia and taste impairment, etc.), a higher proportion of moderate clinical type (31.8 vs. 13.5%, *p* = 0.028) and longer duration of hospitalization (11 vs. 8 days, *p* = 0.027) than those without hyposmia. Whereas, there were no significant differences regarding gender, comorbidity and nucleic acid conversion time between the two groups. Laboratory subgroup analyses demonstrated that patients with hyposmia had slightly low serum IL-6 and TNF-α levels. However, both of the levels were not associated with hyposmia occurrence in multivariate regression analyses. Further follow-up study disclosed that 16 of 22 (72.7%) hyposmia patients had recovered olfaction 1 month later. Serum IL-6 and TNF-α levels were similar between hyposmia recovered patients and those with persistent hyposmia.

**Conclusion:**

Although the incidence of hyposmia after Omicron variant infection is relatively low and the short-term recovery rate is quite high, patients with hyposmia are prone to have a higher proportion of both upper and lower respiratory tract involvements, gastrointestinal and neurological symptoms, contributing to a longer duration of hospitalization.

## Introduction

Novel coronavirus disease 2019 (COVID-19), caused by severe acute respiratory syndrome coronavirus 2 (SARS-CoV-2), has become a pandemic for more than 2 years since December 2019 in Wuhan, China (1). The ongoing COVID-19 pandemic is still a matter of global concern in terms of public health. With the evolution of the virus, Omicron variant, first discovered in southern Africa in November 2021 (2), has replaced the delta variant to become the dominate strain and triggered the fourth wave of COVID-19 worldwide. It also appeared and spread rapidly in Shanghai, China in late February 2022. According to the Shanghai Municipal Health Commission, as of May 4, 2022, more than 600,000 people have been infected, most of them with the fast-spreading Omicron BA.2 variant (3). Clinically, patients infected with COVID-19 Omicron variant had much higher transmissibility, less disease severity and mortality than the previous variants as reported from other countries (4–7).

As one of the neurological manifestations, olfactory dysfunction is a common complaint among COVID-19 patients (8). Hyposmia can be the initial and only symptom during the onset of the disease, and usually shows much improvement within a few weeks in majority of cases (9, 10). Its incidence varies by different virus strains, disease severity and genetic background, ranging from 5.1 to 98.3% (11). It was reported that subjects with older age, Omicron variant, severe clinical classification and East Asian population were associated with low incidence of hyposmia after COVID-19 infection (12, 13). However, infection with Omicron has been rarely examined in East Asia, and only with very small cohorts (14, 15).

The exact pathogenesis of olfactory dysfunction after COVID-19 infection is not fully elucidated. Inflammation of the olfactory system has been reported in COVID-19 related anosmia. Regarding levels of inflammatory markers, Torabi et al. (16) in Iran reported that the pro-inflammatory cytokine, TNF-α level in olfactory epithelium was increased in patients with COVID-19 relative to uninfected controls (16). Experiments have confirmed that virus-infected microglial cells and astrocytes secrete IL-6 and primary glial cells cultured *in vitro* secrete a large number of inflammatory factors, such as IL-6, TNF-α after being infected with coronaviruses (17). In peripheral blood laboratory studies, the results were not consistent. Increased IL-6 levels have been found in serum of patients with hyposmia (18); whereas researchers in Turkey found that serum IL-6 level was lower in patients with COVID-19 related anosmia than those without anosmia (19). Blood tests are easier to obtain than nasal mucosa biopsy. Whether pro-inflammatory cytokines in serum are associated with hyposmia occurrence, severity and recovery of patients with COVID-19 Omicron variant merits investigation.

Therefore, the present study aimed to investigate the incidence, associated clinical characteristics and serum inflammatory parameters associated with olfactory dysfunction in hospitalized non-severe COVID-19 patients with Omicron variant from a Chinese population in Shanghai, China. In addition, the short-term recovery of hyposmia was explored 1 month later *via* telephone interviews.

## Materials and methods

### Subjects

**Figure 1:**
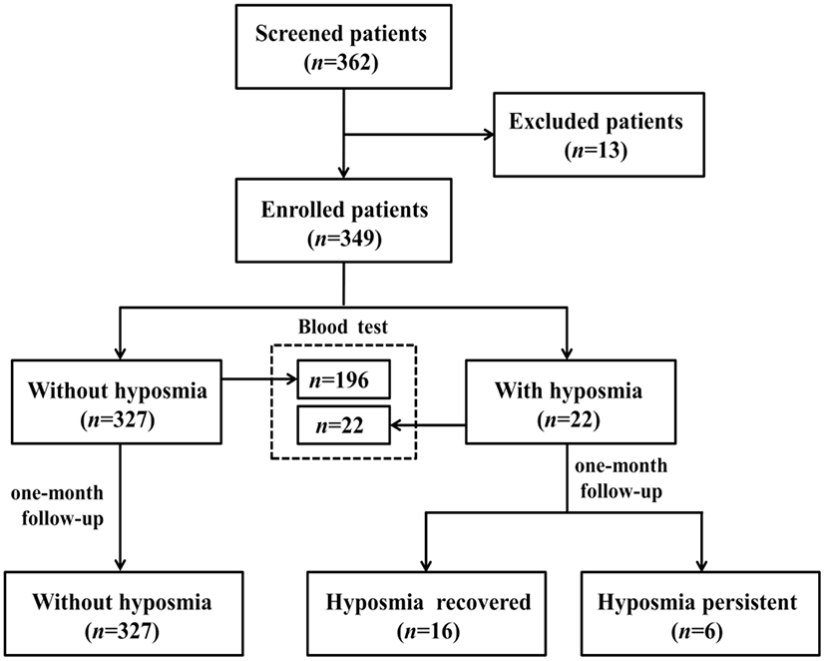
Flow chart of the study.

Between May and June 2022, subjects with non-severe COVID-19 Omicron variant infection admitted in designated hospital of Shanghai Ninth People's Hospital were consecutively screened in this study. All participants were diagnosed with COVID-19 infection according to positive reverse-transcription polymerase chain reaction (RT-PCR) for SARS-CoV2. SARS-CoV-2 viral genomes' phylogenetic characteristics showed that all of the new viral genomes in Shanghai were clustered into the SARS-CoV-2 BA.2.2 sublineage (3). We excluded patients with age under 18 years, pre-existing olfactory dysfunction 1 month before the infection, and obvious cognitive and behavior disorders interfering with further neuropsychological evaluation. We totally screened 362 patients, 13 cases were excluded (1 patient had a history of nasopharyngeal carcinoma, 12 patients could not cooperate to complete the questionnaire). 349 subjects with non-severe COVID-19 Omicron variant infection were enrolled for final analyses (Figure 1). This study was approved by the Medical Ethics Committee of Shanghai Ninth People's Hospital, Shanghai Jiao Tong University School of Medicine.

### Clinical evaluation

A self-designed structural questionnaire was used to obtain related information of the enrolled subjects. Questionnaires were cross-sectionally administered by the doctors working in the general ward of the designated (20) hospital. We collected demographics (age, sex, smoking, etc.), comorbidities (hypertension, diabetes, chronic obstructive pulmonary disease, coronary heart disease, chronic renal disease, etc.), vaccination status and contact history. Clinical symptoms comprising typical (fever, cough, expectoration, sore throat, etc.), gastrointestinal (poor appetite, diarrhea, nausea, vomiting, etc.) and neurological symptoms (fatigue, myalgia, headache, dizziness, taste impairment, etc.) were carefully recorded (Table 1). Olfactory condition was documented for each participant in hospitalization by a face-to-face interview and 1 month later by a telephone interview. According to a research, hyposmia severity was evaluated by visual assessment scale (VAS) ranging from 0 to 100 score (20). The higher the VAS score, the more severe hyposmia the patients had.

**Table 1:** Demographic and clinical characteristics of COVID-19 patients with hyposmia.

**Items**	**Total**	**Without hyposmia**	**With hyposmia**	***p*-Value**
***n***	**349**	**327**	**22**	
Age, years	72.0 (63.0, 82.5)	72.0 (64.0, 84.0)	61.5 (50.7, 71.2)	0.002^**^
Sex				0.120
Male, *n* (%)	158 (45.4)	152 (46.5)	6 (27.3)	
Female, *n* (%)	191 (54.6)	175 (53.5)	16 (72.7)	
Current smoker, *n* (%)	7 (2.0)	6 (1.8)	1 (4.5)	0.369
COVID-19 vaccination status				0.075
Unvaccinated, *n* (%)	208 (59.6)	199 (60.9)	9 (40.9)	
Vaccinated, *n* (%)	141 (40.4)	128 (39.1)	13 (59.1)	
**Comorbidities**				
Any, *n* (%)	214 (61.3)	202 (61.8)	12 (54.5)	0.484
Hypertension, *n* (%)	185 (53.0)	177 (54.1)	8 (36.4)	0.125
Diabetes, *n* (%)	67 (19.2)	67 (20.5)	0 (0)	0.011^*^
COPD, *n* (%)	9 (2.6)	8 (2.4)	1 (4.5)	0.447
Coronary heart disease, *n* (%)	108 (30.9)	103 (31.5)	5 (22.7)	0.480
Chronic renal disease, *n* (%)	57 (16.3)	53 (16.2)	4 (18.2)	0.768
Neurological disease, *n* (%)	92 (26.4)	86 (26.3)	6 (27.3)	1.000
**Symptoms**				
Typical symptoms				
Fever, *n* (%)	156 (44.8)	143 (43.9)	13 (59.1)	0.188
Cough, *n* (%)	252 (72.2)	232 (70.9)	20 (90.9)	0.049^*^
Expectoration, *n* (%)	195 (55.9)	179 (54.7)	16 (72.7)	0.122
Sore throat, *n* (%)	131 (37.5)	117 (35.8)	14 (63.6)	0.012^*^
Runny nose, *n* (%)	106 (30.4)	95 (29.1)	11 (50)	0.053
Nasal obstruction, *n* (%)	57 (16.3)	51 (15.6)	6 (27.3)	0.227
Gastrointestinal symptoms				
Poor appetite, *n* (%)	65 (18.6)	54 (16.5)	11 (50.0)	< 0.001^***^
Diarrhea, *n* (%)	46 (12.3)	35 (10.7)	8 (36.4)	0.002^**^
Nausea, *n* (%)	21 (6.0)	15 (4.6)	6 (27.3)	< 0.001^***^
Vomiting, *n* (%)	13 (3.7)	10 (3.1)	3 (13.6)	0.041^*^
Abdominal pain, *n* (%)	12 (3.4)	11 (3.4)	1 (4.5)	0.548
Neurological symptoms				
Fatigue, *n* (%)	87 (25.0)	78 (23.9)	9 (40.9)	0.123
Myalgia, *n* (%)	72 (20.6)	62 (19)	10 (45.5)	0.006^**^
Headache, *n* (%)	47 (13.5)	41 (12.5)	6 (27.3)	0.097
Dizziness, *n* (%)	46 (13.2)	38 (11.6)	8 (36.4)	0.004^**^
Taste impairment, *n* (%)	19 (5.4)	11 (3.4)	8 (36.4)	< 0.001^**^
Vision impairment, *n* (%)	19 (5.4)	16 (4.9)	3 (13.6)	0.109
Emotional disorder, *n* (%)	17 (4.9)	16 (4.9)	1 (4.5)	1.000
Acute cerebrovascular disease, *n* (%)	3 (0.9)	3 (0.9)	0 (0)	1.000
Impaired consciousness, *n* (%)	2 (0.6)	2 (0.6)	0 (0)	1.000
Seizure, *n* (%)	2 (0.6)	2 (0.6)	0 (0)	1.000
COVID-19 disease classification (admission)				0.430
Asymptomatic or mild, *n* (%)	319 (91.4)	299 (91.4)	20 (90.9)	
Moderate, *n* (%)	30 (8.6)	28 (8.6)	3 (13.6)	
COVID-19 disease classification (discharge)				0.028^*^
Asymptomatic or mild, *n* (%)	298 (85.4)	283 (86.5)	15 (68.2)	
Moderate, *n* (%)	51 (14.6)	44 (13.5)	7 (31.8)	
Clinical treatments				
Oxygen therapy, *n* (%)	40 (11.5)	39 (11.9)	1 (1.5)	0.491
Antiviral-paxlovid, *n* (%)	222 (63.6)	209 (63.9)	13 (59.1)	0.653
Corticosteroids, *n* (%)	58 (16.6)	55 (16.8)	3 (13.6)	1.000
Anticoagulation, *n* (%)	58 (16.6)	55 (16.8)	3 (13.6)	1.000
Antibiotic, *n* (%)	59 (16.9)	53 (16.2)	6 (27.3)	0.234
Intravenous immunoglobulin, *n* (%)	8 (2.3)	7 (2.1)	1 (4.5)	0.409
Thymosin, *n* (%)	72 (20.6)	69 (21.1)	3 (13.6)	0.587
Nutritional support, *n* (%)	106 (30.4)	100 (30.6)	6 (27.3)	0.477
Clinical outcomes				
Duration of Hospitalization	8 (5, 11)	8 (5, 11)	11 (7, 13)	0.027^*^
Turning to nucleic acid negative duration	10 (7, 13)	10 (7, 13)	10 (8, 12)	0.901

* p < 0.05;

** p < 0.01;

*** p < 0.001.

Chest CT scan, clinical treatment (oxygen therapy, corticosteroids, anticoagulation, antibiotic, nutritional support, etc.) and outcomes (duration of hospitalization, time period until the nucleic acid amplification test turned negative, transfer to Intensive Care Unit, death, etc.) were also recorded. Disease classification was determined as asymptomatic, mild, moderate, severe and critical condition, according to the ninth version of Chinese COVID-19 diagnosis and treatment protocol for COVID-19 patients (21). Patients with typical pneumonia changes on CT such as patchy ground-glass opacities were classified into moderate subtype.

### Biochemical analyses

To explore the biochemical parameters associated with COVID-19 related hyposmia, two hundred and eighteen patients with detailed biochemical information were enrolled as a subgroup.

Routine blood biochemistry including total white blood cell (WBC), neutrophil, lymphocyte and monocyte count, percentages of neutrophil and lymphocyte hemoglobin, platelet count, C-reactive protein (CRP), coagulation function including prothrombin time (PT), activated partial thromboplastin time (APTT), fibrinogen and D-dimer were analyzed during hospitalization. In addition, two pro-inflammatory cytokines in serum, IL-6 and TNF-α were measured in this subgroup of 218 cases.

### Statistical analyses

SPSS version 23.0 (IBM Corporation, Armonk, NY, USA) was used for statistical analysis. Continuous variables are expressed as the means ± SD or medians [interquartile ranges (IQR), Q1–Q3]; categorical variables are expressed as frequencies and percentages. Comparisons of means between the two groups were performed using the independent *t*-test or the Mann-Whitney U test as appropriate. To compare categorical data among groups, we applied the chi-square test or Fisher's exact test. Two linear regression analyses were used to explore the independent associated factors of serum IL-6 and TNF-α levels, respectively. B value and 95% confidence intervals (CIs) were reported accordingly. The test level (α) was set at 0.05.

## Results

### Incidence, demographic and clinical characteristics of COVID-19 patients with hyposmia

Among the enrolled 349 cases infected with COVID-19 Omicron variant, 22 patients had hyposmia during hospitalization. So, the prevalence of hyposmia in this cohort was 6.3%. The mean VAS score of these patients with hyposmia was 54.8 ± 25.3 points.

Demographically, COVID-19 patients with hyposmia were younger than those without hyposmia (61.5 vs. 72.0, *p* = 0.002, Table 1). There was no statistically significant difference in gender between the two groups. There was a trend that patients with hyposmia had a marginal increase of vaccination rate (59.1 vs. 39.1%, *p* = 0.075, Table 1) relative to those without hyposmia. Although none of the patients with hyposmia had diabetes, the number of comorbidities was similar between the two groups. Regarding clinical symptoms, patients in the hyposmia group had more typical [cough (90.9 vs. 70.9%, *p* = 0.049) and sore throat (63.6. vs. 35.8%, *p* = 0.012)], gastrointestinal [(poor appetite (50.0 vs. 16.5%, *p* < 0.001), diarrhea (36.4 vs. 10.7%, *p* = 0.002), nausea (27.3 vs. 4.6%, *p* < 0.001) and vomiting (13.6 vs. 3.1%, *p* = 0.041)] and neurological [myalgia (45.5 vs. 19.0%, *p* = 0.006), dizziness (36.4 vs. 11.6%, *p* = 0.004) and taste impairment (36.4 vs. 3.4%, *p* < 0.001)] symptoms, in comparison with those without hyposmia (Table 1).

**Figure 2:**
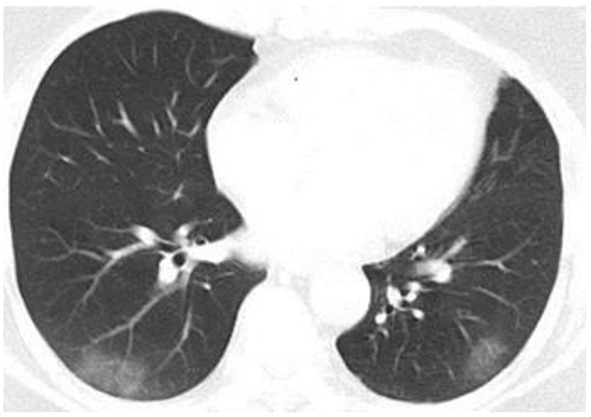
Chest CT image of a COVID-19 patient with hyposmia. Axis chest CT scan showed bilateral patchy ground-glass opacities consistent with typical moderate COVID-19.

Concerning COVID-19 severity, patients in the hyposmia group had a higher proportion of moderate COVID-19 (31.8% vs. 13.5%, *p* = 0.028) at discharge relative to those without hyposmia (Table 1), indicating a higher proportion of lung involvement in this subgroup (Figure 2). Although the clinical treatment and time period of conversion of the nucleic acid amplification test from positive to negative were similar, COVID-19 patients with hyposmia had longer duration of hospitalization (11 vs. 8 days, *p* = 0.027, Table 1). None of the enrolled subjects were transferred to intensive care unit (ICU) or died.

### Laboratory analysis of COVID-19 patients with hyposmia

Subgroup analysis based on 218 patients (Figure 1) demonstrated that subjects with hyposmia had slightly lower serum IL-6 (3.71 vs. 6.11, *p* < 0.001) and TNF-α (5.72 vs. 9.34, *p* = 0.010) levels than those without hyposmia (Table 2). There was no statistical difference in terms of blood routine, coagulation function, C-reactive protein and other inflammatory indicators between the two groups. However, linear regression analyses demonstrated that older age was independently associated with IL-6 levels; also, older age and diabetes were independently associated with TNF-a levels in serum (Table 3).

**Table 2:** Laboratory findings of COVID-19 patients with hyposmia (*n* = 218).

**Laboratory finding**	**Without hyposmia**	**With hyposmia**	***p*-Value**
***n***	**196**	**22**	
White blood cell count, ^*^10^∧^9/L	5.29 ± 1.78	5.25 ± 2.36	0.235
Neutrophil cell count, ^*^10^∧^9/L	2.97 (2.09, 4.14)	2.31 (1.65, 3.64)	0.197
Lymphocyte count, ^*^10^∧^9/L	1.30 (1.00, 1.70)	1.60 (1.00, 2.10)	0.107
Monocyte count, ^*^10^∧^9/L	0.51 (0.39, 0.67)	0.50 (0.42, 0.72)	0.940
Hemoglobin, g/L	130.25 ± 17.53	128.23 ± 16.11	0.223
Platelet count, ^*^10^∧^9/L	187.95 ± 66.36	208.59 ± 78.05	0.268
Neutrophil/ Lymphocyte	1.95 (1.44, 3.51)	1.48 (1.25, 2.20)	0.052
Monocyte / Lymphocyte	0.39 (0.27, 0.57)	0.38 (0.25, 0.46)	0.178
C-reactive protein, mg/L	3.81 (1.58,10.30)	2.29 (0.68, 6.60)	0.121
PT, s	10.90 (10.40, 11.40)	10.60 (10.40, 11.10)	0.097
APTT, s	28.50 (26.50, 30.50)	28.60 (26.88, 30.12)	0.808
Fibrinogen, g/L	3.04 (2.58, 3.60)	2.90 (2.55, 3.29)	0.427
D-dimer, mg/L	0.42 (0.22, 0.79)	0.26 (0.13, 1.29)	0.398
IL-6, pg/mL	6.11 (4.01, 9.11)	3.71 (0.00, 4.81)	< 0.001^***^
TNF-α, pg/mL	9.34 (6.73, 12.13)	5.72 (0.00, 8.01)	0.010^**^

* p < 0.05;

** p < 0.01;

*** p < 0.001.

**Table 3:** Independent associated factors of serum IL-6 and TNF-α levels in COVID-19 patients (*n* = 218).

**Items**	**Univariate regression**		**Multivariate regression**	
	***B* (95%CI)**	***p*-value**	***B* (95%CI)**	***p*-value**
**Model 1: Dependent factor: serum IL-6 level**				
Age: > 72 years	5.96 (3.34, 8.58)	< 0.001^***^	5.40 (2.47. 8.32)	< 0.001^***^
COVID-19 disease severity: Moderate	2.03 (−1.73, 5.79)	0.288	0.36 (−3.38, 4.01)	0.849
Vaccination status: Unvaccinated	3.00 (0.26, 5.74)	0.032^*^	1.26 (−1.58, 4.10)	0.383
With diabetes	2.68 (−0.68, 6.04)	0.117	1.76 (−1.57, 5.09)	0.299
With hyposmia	−1.22 (−6.18, 3.74)	0.630	1.29 (−3.68, 6.26)	0.609
**Model 2: Dependent factor: serum TNF-α** **level**				
Age: > 72 years	3.81 (2.28, 5.35)	< 0.001^***^	2.79 (1.12, 4.46)	0.001^**^
COVID-19 disease severity: Moderate	1.20 (−1.01, 3.42)	0.286	0.80 (−1.34, 2.93)	0.462
Vaccination status: Unvaccinated	2.03 (0.42, 3.65)	0.014^*^	0.80 (−0.82, 2.43)	0.331
With diabetes	3.57 (1.62,5.52)	< 0.001^***^	2.70 (0.78, 4.61)	0.006^**^
With hyposmia	−4.10 (−6.91, −1.30)	0.004^**^	−2.44 (−5.22, 0.35)	0.087

CI, confidence interval;

* p < 0.05;

** p < 0.01;

*** p < 0.001.

We also did a correlation analysis between those two cytokines and hyposmia VAS score. It revealed that neither IL-6 (*r* = −0.022, *p* = 0.929) nor TNF-a (*r* = −0.008, *p* = 0.974) levels in serum were related to hyposmia severity.

### Olfactory recovery of COVID-19 patients with hyposmia at one-month follow-up

COVID-19 patients with hyposmia (*n* = 22) were followed up by telephone interviews 1 month after discharge (Figure 1). Olfactory function still did not return to normal in 6 of 22 patients (27.3%). Subsequently, we compared the differences in baseline VAS scores of hyposmia and laboratory indicators between the hyposmia recovered (*n* = 16) and persistent group (*n* = 6). It demonstrated that there were no significant differences in terms of initial VAS score (52.2 ± 25.7 vs. 61.7 ± 24.8, *p* = 0.528), serum IL-6 (3.56 vs. 4.46, *p* = 0.803) or TNF-α (5.07 vs. 7.83, *p* = 0.184) levels between hyposmia recovered and persistent groups.

Altogether, our results suggested that although there was a slight reduction of serum IL-6 and TNF-α levels in the hyposmia group, both of the two pro-inflammatory cytokine levels in serum were not associated with hyposmia occurrence, severity or recovery in COVID-19 patients. Aging and diabetes may influence the expression of the two cytokines in serum.

## Discussion

To our knowledge, this is the first study in China reporting the epidemiological data of olfactory dysfunction after Omicron variant infection. Our results based on 349 patients with non-severe COVID-19 Omicron variant enrolled in designated hospital revealed that (1) the incidence of hyposmia after Omicron infection was relatively low and the short-term recovery rate was quite high; (2) patients with hyposmia had more associated clinical symptoms and increased proportions of both upper and lower respiratory tract involvements, contributing to a longer duration of hospitalization;(3) serum IL-6 and TNF-α levels were not related to hyposmia occurrence, severity or recovery.

We validated that Chinese COVID-19 patients with Omicron variant also had relatively low hyposmia incidence. This finding was consistent with the reports from other countries. Loss of smell was less likely among people infected during Omicron prevalence than during delta prevalence, according to a ZOE COVID study conducted in the UK (16·7 vs 52·7%) (4). A systematic review based on the first 12 reports revealed that approximately 13% of patients with Omicron infection had involvement of Smell (22), which was 3–4-fold lower than the prevalence in times and regions when the alpha and delta variants prevailed. All these findings indicate that Omicron variant largely spares the olfactory function. In comparison to earlier strains, the new mutations make Omicron more hydrophobic and alkaline, which may lessen mucus layer penetration. Omicron very slightly alters receptor binding affinity, however, entry efficiency into host cells is reduced in cells expressing the TMPRSS2 protease. The sustentacular cells in the olfactory epithelium, which are the novel Omicron variant's primary target cells, may be less likely to become infected because they abundantly express TMPRSS2. In addition, genetic background may also contribute to the low incidence of hyposmia in Chinese population (12). Shelton et al. reported that the UGT2A1/UGT2A2 locus was associated with COVID-19-related loss of smell or taste, which differed significantly between ethnicities (23). All these factors may explain the low incidence and high recovery of hyposmia in our cohort. Compared with those in Western Countries, patients in East Asia had less olfactory impairment. During the battle against COVID-19 in the past 2 years, more and more residents received COVID-19 vaccination in China. The usefulness of vaccination in reducing the severity of COVID-19 has been adequately proven (24); however, there is not enough evidence to establish a link between vaccination and the low occurrence of chemosensory disorders (25).

A novel finding is that patients with hyposmia had more upper respiratory (sore throat), lower respiratory (cough), gastrointestinal (poor appetite, diarrhea, nausea and vomiting) and neurological (myalgia and taste impairment) symptoms as demonstrated by our detailed symptomatic descriptions. Also, such kind of patients were more likely to have lung infiltration as revealed by COVID-19 clinical classification. All these factors could result in a possible longer hospital stay. Burges Watson et al. in Italy reported that COVID-19 patients with hyposmia also had a higher proportion of altered eating, appetite loss and weight changes (26). Smell and taste impairments are typical chemosensory dysfunctions, and usually correlated to each other after COVID-19 infection (27). It was also reported that COVID-19 related myalgia was a risk factor for persistent hyposmia (28). Although higher proportions of lung infiltration at discharge in patients with hyposmia were found, none of them had converted to severe/critical stage, indicating the pulmonary infiltration in such kind of patient is not severe. The underlying mechanism is still unknown. Some previous studies found that hyposmia appeared less in severe COVID-19 patients and may represent a favorable prognosis (29). Our study suggested that hyposmia could be a marker indicating high proportions of both upper and lower respiratory involvements. Hyposmia in COVID-19 patients may not be as benign as reported. This has important clinical implications. For these patients, more attention should be paid to their pulmonary conditions. Close monitoring and active treatment are required.

The pathogenesis of hyposmia related to COVID-19 is still not fully elucidated. Accumulating evidence suggested that pro-inflammatory cytokines, IL-6 and TNF-α may be associated with hyposmia secondary to COVID-9 infection. We found the serum IL6 and TNF-α levels were not correlated with hyposmia occurrence, severity or recovery, which was consistent with Sanli's report in Turkey (19) and Vaira's report in Italy (30). Regarding nasal biopsies, Torabi et al. (16) reported that the pro-inflammatory cytokine, TNF-α level in olfactory epithelium was increased in patients with COVID-19 relative to uninfected controls (16). One autopsy study in two patients found that there was inflammatory olfactory neuropathy, mainly axonal damage in olfactory epithelium in two patients with COVID-19 (31), whereas, the olfactory tracts were largely unremarkable. Significant pathology in central nervous system structures, including those related to olfaction, appears to be relatively rare (32). Based on these results, we infer that local inflammation in nasal mucosa rather than the systemic inflammation may contribute to COVID-19 related hyposmia in the acute stage (33). More mechanism research of COVID-19 related hyposmia is warranted in future.

In our study, 6 of 22 patients (27.3%) still had olfactory deficits at one-month follow-up. Whether their persistent hyposmia will develop into long-term sequelae merits investigation. Possibly, COVID-19 patients with persistent hyposmia had affections of a larger area of the sensory epithelium, presumably with more extensive epithelium damage that resulted in the loss of more olfactory receptor neurons (12). The notion of the SARS-CoV-2 virus being neurotropic in humans and invading the brain through the olfactory nerve is highly controversial (34). A more extensive viral propagation into other brain regions would have been expected if systemic hematogenous involvement occurred. However, other studies have suggested that olfactory transmucosal virus invasion is a port of central nervous system entry in individuals with COVID-19 (35). Neurodegenerative diseases may be accelerated by an inflammatory signal from the nasal olfactory epithelium to the olfactory bulbs and associated brain areas. Long-term longitudinal follow-up is needed to explore the association between persistent olfactory dysfunction and phenotypic conversion of neurodegenerative diseases (36), such as Parkinson's disease.

This study has a few limitations. First, severe/critical patients were not included in the study, since the proportion of such kind of patients was relatively low, and they were usually transferred to ICU, not treated in general ward. Second, we used subjective VAS score to evaluate the occurrence and severity of hyposmia, which may underestimate the real hyposmia incidence compared with objective evaluation method such as sniffin' sticks. Third, the sample size of patients with hyposmia is relatively small. The findings are exploratory. Multi-center registry studies for patients with hyposmia are needed in future.

## Conclusion

Our study based on Chinese population broadens the epidemiological data and phenotypic characteristics of Omicron related hyposmia. Although the incidence of hyposmia after Omicron infection is relatively low and the short-term recovery is quite high, patients with hyposmia are prone to have higher proportions of both upper and lower respiratory tract involvements, gastrointestinal and neurological symptoms, contributing to longer hospitalized duration. COVID-19 with hyposmia may not be as benign as reported. Close monitoring and active treatment are needed for such kind of patients. Systematic inflammation in serum may not contribute to COVID-19 related hyposmia in the acute stage. More mechanism research and long-term follow-up of hyposmia in COVID-19 are warranted in future.

## Data availability statement

The original contributions presented in the study are included in the article/supplementary material, further inquiries can be directed to the corresponding author.

## Ethics statement

The studies involving human participants were reviewed and approved by the Medical Ethics Committee of Shanghai Ninth People's Hospital, Shanghai Jiao Tong University School of Medicine, Shanghai, China (2022-T130-2). Written informed consent for participation was not required for this study in accordance with the national legislation and the institutional requirements.

## Author contributions

JS, LW, WC, and JL had a role in the design of the study. JS, LW, XS, PW, YJ, WC, and JL had a role in the acquisition and interpretation of data. LW and WC analyzed the data and interpreted it. JS, LW, and WC drafted the manuscript. WC revised it. The final version of the manuscript was amended with input from all authors, who also gave their approval.

## Funding

This study was supported by grants from 200 talent project from Shanghai Municipal Education Commission-Gaofeng Clinical Medicine Grant Support (20161422), Natural Science Foundation Project from the Shanghai Municipal Science and Technology Commission (22ZR1436900), Clinical Research Program of Shanghai Ninth People's Hospital Affiliated to Shanghai Jiao Tong University School of Medicine (JYLJ202003), and Project of Biobank from Shanghai Ninth People's Hospital Affiliated to Shanghai Jiao Tong University School of Medicine (YBKB202120).

## Conflict of interest

The authors declare that the research was conducted in the absence of any commercial or financial relationships that could be construed as a potential conflict of interest.

## Publisher's note

All claims expressed in this article are solely those of the authors and do not necessarily represent those of their affiliated organizations, or those of the publisher, the editors and the reviewers. Any product that may be evaluated in this article, or claim that may be made by its manufacturer, is not guaranteed or endorsed by the publisher.
